# The ethanol extract of *Edgeworthia gardneri* (Wall.) Meisn attenuates macrophage foam cell formation and atherogenesis in ApoE^−/−^ mice

**DOI:** 10.3389/fcvm.2022.1023438

**Published:** 2022-11-24

**Authors:** Le Tang, Cuifang Kuang, Dan Shan, Min Shi, Jiangsheng Li, Liang Qiu, Jun Yu

**Affiliations:** ^1^Centre for Translational Medicine, Jiangxi University of Chinese Medicine, Nanchang, China; ^2^Jiangxi Key Laboratory of Traditional Chinese Medicine for Prevention and Treatment of Vascular Remodeling Diseases, Jiangxi University of Chinese Medicine, Nanchang, China; ^3^Department of Cardiovascular Sciences and Centre for Metabolic Disease Research, Lewis Katz School of Medicine, Temple University, Philadelphia, PA, United States

**Keywords:** *Edgeworthia gardneri* (Wall.) Meisn, macrophage, foam cell, atherosclerosis, CD36

## Abstract

**Introduction:**

Atherosclerotic cardiovascular disease is the leading cause of death worldwide. The *Edgeworthia gardneri* (Wall.) Meisn is a Tibetan medicine commonly used to prepare herbal tea to alleviate the local people's metabolic diseases. However, the anti-atherosclerotic effect of ethanol extract of the flower of *E. gardneri* (Wall.) Meisn (EEEG) and its underlying mechanism remain unknown.

**Methods:**

EEEG was used to treat low-density lipoprotein (ox-LDL)-induced macrophages to detect macrophage foaminess, cholesterol binding and uptake, and lipid transport-related gene expression. eEEG treated ApoE^−/−^ mice fed a high-fat diet for 16 weeks to detect atherosclerotic plaque area, macrophage infiltration, and liver and small intestine lipid transport-related gene expression.

**Results:**

EEEG inhibited macrophage-derived foam cell formation induced by oxidized low-density lipoprotein (ox-LDL) by reducing CD36-mediated lipoprotein uptake. EEEG significantly alleviated atherosclerosis in ApoE^−/−^ mice fed a high-fat diet for 16 weeks. EEEG treatment significantly decreased atherosclerotic plaque area, macrophage infiltration, and increased collagen content. Moreover, EEEG treatment significantly downregulated mRNA expression of hepatic *Srb1* and intestinal *Npc1l1* and increased expression of hepatic *Cyp7a1*.

**Conclusion:**

Our study highlighted that EEEG played a role in attenuating atherosclerotic plaque formation by reducing macrophage foam cell formation.

## Introduction

According to the World Health Organization statistics, cardiovascular disease will affect 23.6 million people worldwide by 2030. Atherosclerosis (AS) has become the leading cause of death in patients with cardiovascular disease and the primary pathological basis of cardiovascular disease. The pathogenic mechanism underlying AS is complex and closely related to abnormal lipid metabolism, vascular endothelial injury, genetic factors, and hemodynamic changes. At present, antioxidant, lipid-regulating, and antiplatelet drugs are commonly used to treat AS. Lipid-lowering drugs such as statins, fibrates, ezetimibe, and PSCK9 inhibitors are mainly used to prevent and treat AS in clinical practice. Although they slow down the disease progression, these drugs can only reduce the mortality of cardiovascular diseases by 30% and may lead to complications and adverse reactions, such as respiratory tract infection, muscle pain, low back pain, joint pain, and other side effects caused by ezetimibe (1). Given the adverse reactions associated with current AS management, safer and more effective anti-atherosclerotic drugs are urgently needed.

In the process of AS occurrence, oxidized low-density lipoprotein (ox-LDL) is continually uptaken by macrophages. When the intracellular cholesterol far exceeds the scavenging capacity, significant cholesterol will accumulate in the macrophages and transform them into foam cells (1). The foam cell formation is a fundamental step in initiating and developing atherosclerosis plaque (2). The scavenger receptors SR-A1, SR-B1, and CD36 expressed in the macrophage are responsible for binding to and taking up ox-LDL. ATP-binding cassette transporters like ABCA1 and ABCG1 are members of the ABC superfamily of transmembrane transporters, which mediate cholesterol efflux to apolipoproteinA1 (apoA1) and high-density lipoprotein (HDL) (3). Therefore, attempts to reduce foam cells may be a potential therapeutic strategy for inhibiting early-stage atherosclerosis pathogenesis (4).

The *Edgeworthia gardneri* (Wall.) Meisn (EG) belongs to Family_Thymelaeaceae Genus_*Edgeworthia Meisn*. The dry flower bud of EG is a widely recognized Tibetan medicine because of its long-term use, pollution-free drug source, and unique efficacy. It is known as one of the “Eighteen Treasures of Qinghai Tibet” in China. The flower of EG contains flavonoids, polysaccharides, volatile oils, fatty acids, triterpenes, and nitrogen-containing compounds. It has been used to prepare herbal tea to alleviate metabolic diseases (5). Previous researches have shown that EG has anti-hyperglycemia, anti-insulin resistance, and anti-adipogenesis activities, and the underlying mechanisms may be related to α-glucosidase and α-amylase inhibition, IRS1/GSK3β/FoxO1 and PPARγ/β signaling pathway activation, and gut microbiota modulation (6–10). However, the anti-atherosclerotic potential effect of EG and its underlying mechanism have not been reported.

Here, we evaluated the effect of ethanol extract of flower of *Edgeworthia gardneri* (EEEG) on atherosclerosis in an ApoE^−/−^ mice fed a high-fat diet (HFD). The effect and mechanism of EEEG on promoting reverse cholesterol transport (RCT) and inhibiting foam cell formation were also investigated.

## Materials and methods

### Materials and chemicals

The EG was purchased from Zangxi Tang (Tibet, China), and a voucher specimen (No.GH827) was deposited in the Jiangxi University of Chinese Medicine, Nanchang, China. MTT (M1020), Dimethyl sulfoxide (D8370), Trypsin (T8150), Oil red O powder (O8020), high sugar Dulbecco's modified Eagle's medium (DMEM) (12100-500), and PMI 1640 medium (31,800) were purchased from Solarbio (Beijing, China). Fetal Bovine Serum (FBS) (CC-4101A) was purchased from Lonza (Walkersville, MD, USA). Human oxidized low-density lipoprotein (ox-LDL, yb-002) and fluorescently labeled oxidized low-density lipoprotein (yb-0010) were obtained from Yiyuan Biology (Guangzhou, China). Bodipy TM493/503(D3922)was purchased from Invitrogen (Carlsbad, CA, USA). Rat anti-mouse CD68 antibody (MCA1957) or Monoclonal anti-alpha-smooth muscle-FITC antibody (F3777) was purchased from Bio-Rad (Kidlington, USA) or Sigma-Aldrich (St. Louis, MO, USA). Rabbit Polyclonal Anti-CYP7A1 antibody (TA351400) was purchased from Origene. Blood lipid test kits were purchased from Nanjing Jiancheng Bioengineering Institute.

### Animals and treatment

Forty-six 6–8-week-old male ApoE^−/−^ mice (20–22 g) were purchased from Nanjing Biomedical Research Institute of Nanjing University [Certificate of Conformity No. SCXK (Su) 2015-0001]. Mice were housed in IVC cages (temperature 20–26°C, humidity 40–70%) under alternating 12 h light and 12 h dark conditions and given adequate food. The animal experiments were approved by the Ethics Review Committee of the Jiangxi University of Chinese Medicine. Mice fed a high-fat diet were randomly divided into four groups (*n* = 11 or 12): HFD group treated with 100 μL sterile PBS (0.01 M); HFD group treated with low (1 g/kg of body weight), medium (2 g/kg of body weight), and high doses (4 g/kg of body weight) of EEEG solution, respectively. All mice were administered orally with PBS or EEEG once a day for 16 weeks and then sacrificed after fasting for 8 h. Blood samples were collected. The full-length aorta and heart specimens were fixed in 4% PFA.

### Preparation of *Edgeworthia gardneri* ethanol extract

The EG (2.7 kg) was extracted three times with 60% ethanol (total volume 9 L) for 2 h. The combined extracts were concentrated under reduced pressure and extracted with petroleum ether, petroleum ether extracts were obtained, and the residue was partitioned into H_2_O and extracted with 30% ethanol. After that, 30% ethanol extract was obtained. Then the 30% EEEG was dissolved in dimethyl sulfoxide (DMSO) and PBS to get a stock solution for cell and animal experiments, respectively.

The EEEG was qualitatively and quantitatively analyzed by HPLC using a Waters Acquity TM Ultra Performance LC system (Waters Corporation, Milford, MA, USA) in conjunction with a Waters HSS T3 TM (150 × 2.1 mm, 1.8 μm) column. The column temperature was maintained at a constant 25°C. The mobile phase flow rate was 0.8 ml/min. The mobile phase consists of acetonitrile (solvent A) and H_2_O (solvent B), and both A and B contain 0.1% methanol. The elution procedure was set as performed as described before (10): 0–1 min, 1% A; 1.1–8 min, 1% A; 8.1–10 min, 99% A; and 10.1–12 min, 1% A. Results were shown in Supplementary Figure 1.

### Isolation of mouse bone marrow-derived macrophages

L929 cells were cultured in RPMI 1640 medium and incubated in a humidified atmosphere (5% CO_2_; 37°C) for 5 days. The culture media was then collected and centrifuged at 1,000 rpm for 5 min, and the supernatant was harvested as the L929 conditioned medium.

Eight-week-old male C57BL/6 mice were sacrificed and immersed in 75% ethanol solution for 5 min for sterilization. Bilateral femurs were separated and washed in the macrophage starvation medium. Then the ends of the femurs were cut off, and the bone marrow in the femurs was flushed out with DMEM. Cells were centrifuged at 3,000 rpm for 5 min and suspended in DMEM supplemented with 10% fetal bovine serum, 100 U/mL penicillin, and 100 U/mL streptomycin. Cells were then placed in 10-cm dishes and incubated in a humidified atmosphere (5% CO_2_, 37°C) for 72 h. Floating cells in the medium were collected and centrifuged. The harvested cells were resuspended with an L929 conditioned medium and adhered to the cell dish, followed by replacing the medium with a fresh medium.

### BMDM viability assay

BMDMs were transferred into 96-well-plates (4 × 10^4^ cells/well) and incubated for 5 days. Then, 200 μL of medium containing different concentrations of EEEG (0.1, 1, 10, 100, and 200 μg/ml) was added to each well and incubated for 24 h. After washing twice with PBS, 200 μL of MTT solution (1 mg/mL) was added to each well and incubated for 4 h. Finally the MTT solution was removed, followed by adding 100 μL of DMSO to each well and incubated for 10 min. The absorbance of all samples was measured at 490 nm.

### Foam cell formation assay

Cells or full-length aortas fixed in 4% paraformaldehyde (PFA) solution were washed three times with PBS, rinsed with the 60% isopropyl alcohol solution for 1 min, and stained with freshly prepared Oil red O working solution for 15 min. Cells or full-length aortas were rinsed briefly with 60% isopropyl alcohol and carefully rinsed with distilled water. Then, cells or full-length aortas were observed under the microscope and photographed. The average Oil red O positive areas were calculated relative to the number of cells.

Raw 264.7 cells were inoculated in the 24-well-plates (1 × 10^5^ cells/well) and incubated at 37°C for 24 h. Then, the Raw 264.7 cells were treated with 80 μg/ml of ox-LDL for 24 h, followed by washing twice with PBS. 200 μL of BODIPY working solution (1 μg/ml) was added to each well, incubated in the dark for 20 min at 37°C, and washed with acidic PBS (pH 2.7; 25 mM Glycine; 3% BSA) for 5 min. After that, Raw 264.7 cells were fixed in 4% PFA solution for 15 min and washed three times with PBS, followed by staining with DAPI (1:1,000) staining solution for 3 min and washing with PBS for 3 times. Cells were observed by laser confocal fluorescence microscopy and photographed. The average fluorescence intensity was quantified by Image J and calculated as the total area of green fluorescence intensity relative to the number of cells.

THP-1 cells (human myeloid leukemia mononuclear cells) were cultured in the 24-well-plates (3 × 10^5^ cells/well) in 1640 medium, and cells were incubated with 80 μg/ml ox-LDL for 24 h, simultaneous intervention with 1 μM rosiglitazone and intervention with 1 μg/mL EEEG for 24 h. Cells fixed in 4% paraformaldehyde (PFA) solution were washed three times with PBS, rinsed with the 60% isopropyl alcohol solution for 1 min, and stained with freshly prepared Oil red O working solution for 15 min. Cells were rinsed briefly with 60% isopropyl alcohol and carefully rinsed with distilled water. Then, cells were observed under the microscope and photographed. The average Oil red O positive area was calculated as the total area of Oil red O relative to the number of cells.

### Analysis of Dil-oxLDL binding and uptake

Binding assay: BMDM cells which covered the bottom of 12-well-plates were stimulated with EEEG and Dil-oxLDL at 4°C for 30, 60, 90, and 120 min, respectively (11). Uptake assay: BMDM cells were stimulated with EEEG and Dil-oxLDL for 2, 4, and 6 h, respectively (11).

Cells were washed 4 times with acidic PBS for 5 min, digested with trypsin for 5 min, and centrifuged at 1,000 rpm for 3 min. Cell pellets were suspended in PBS, transferred to the sample tube, and analyzed by flow cytometry.

### Histopathological staining and immunofluorescence analysis

The collected aortic roots fixed in 4% PFA were washed with PBS. The samples were then embedded in an optimal cutting temperature compound, frozen in liquid nitrogen, and cut into serial 10 μm-thick cryosections from the aortic root to the apex. A series of sections were collected on a stereomicroscope slide and stained with Oil red O, hematoxylin-eosin, and Masson's trichrome (Solarbio, China).

The sections stained with Oil red O were counterstained with hematoxylin-eosin for 30 s. After washing with tap water for 2 min, the sections were mounted with glycerin and gelatin and photographed using Nikon 4,500 digital camera. The sizes of atherosclerotic plaque and collagen fibers were determined using Image J software.

Frozen sections rinsed with PBS solution for 15 min were blocked in a solution containing 5% donkey serum, 0.5% bovine serum albumin (BSA), and 0.03% Triton X-100 for 1 h and the sections were incubated with primary anti-CD68 (1:250) or anti-α-SMA mouse antibody (1:500) overnight at 4°C, respectively. After rinsing with PBS solution for 15 min, the sections were incubated with fluorescence-conjugated secondary antibody for 1 h and stained with 4,6-diamidino-2-phenylindole (DAPI) for 3 min. After removing the DAPI solution, sections were photographed using fluorescence microscopy, and the average fluorescence intensity was quantified by Image J and calculated as the total area of fluorescence intensity relative to the plaque area.

### Quantitative RT-PCR

Total RNA from cells or tissues was extracted using Trizol reagents. cDNA was synthesized using PrimeScriptTM RT reagent Kit (Takara, Kyoto, Japan) following the manufacturer's instructions. Real-time PCR was performed following the SYBR® Premix Ex TaqTM II (Takara, Kyoto, Japan). The primer sequences are shown in Supplementary Table 1. β*-actin* was used as the internal control.

### Western blotting

Tissues were homogenized with 150 μl of RIPA lysis buffer. The lysis buffer was then centrifuged (12,000 rpm, 15 min, 4°C). Protein was quantified in the supernatant using a BCA protein assay kit. The primary antibodies of CYP7A1 (TA351400, Origene), were used. Approximately 40 μg of total protein was resolved by sodium dodecyl sulfate-polyacrylamide gel electrophoresis, electroblotted onto 0.45-μm polyvinylidene fluoride membranes, and probed overnight at 4°C. Membranes were incubated with secondary horseradish peroxidase-antibodies (anti-mouse or anti-rabbit) for 1 h at room temperature. Immunoblots were detected with Image Studio.

### Determination of serum lipid profiles

Lipid profiles, including total cholesterol (TCHO), triglycerides (TG), low-density lipoprotein-cholesterol (LDL-C), and high-density lipoprotein-cholesterol (HDL-C) were measured using commercially available kits with a multifunctional enzyme marker according to the manufacturer's instructions.

### Statistical analysis

All values were expressed as the mean ± S.E.M and analyzed using GraphPad Prism 9.0.2 software (San Diego, CA, USA). One-way analysis of variance (ANOVA) followed by Dunnett's test was used to evaluate statistical differences among groups. A value of *P* < 0.05 described a statistically significant difference.

## Results

### EEEG inhibits ox-LDL-induced macrophage foam cell formation

**Figure 1:**
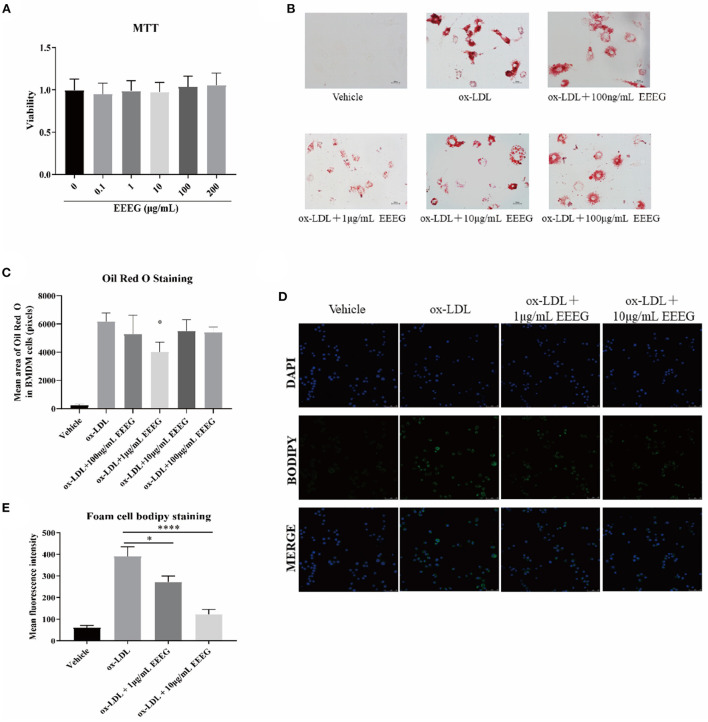
EEEG treatment reduces macrophage foam cell formation. **(A)** Effect of EEEG treatment on BMDM viability. **(B)** Foam cell formation assayed by oil-red-O staining. Cells were incubated for 24 h with ox-LDL in the presence or absence of EEEG, followed by Oil red O staining. **(C)** Quantification of ox-LDL uptake by macrophages. **(D)** BODIPY staining of RAW 264.7 cells. Cells were incubated for 24 h with ox-LDL in the presence or absence of EEEG, followed by BODIPY staining. **(E)** Quantification of BODIPY fluorescence staining. Data are expressed as mean ± s.e.m., Statistical analysis was based on Graphpad Prism 9.0.2 software and a value of *P* < 0.05 was considered statistically significant. One-way ANOVA with Student Neuman-Keuls *post-hoc* test was performed to compare the data between multiple groups, *p < 0.05, ****p < 0.0001 vs. ox-LDL.

To determine the non-cytotoxic dose range, freshly isolated mouse BMDMs were treated with different concentrations (0.1, 1, 10, 100, and 200 μg/mL) of EEEG for 24 h. As shown in Figure 1A, EEEG did not induce cell death at all concentrations tested. The concentrations of 100 μg/mL or lower were used for all the following experiments. Macrophages can uptake the excess lipids, differentiate into lipid-laden foam cells, and promote the progression of atherosclerosis (12). To investigate the effect of EEEG on macrophage foam cell formation, BMDM cells were pretreated with vehicle, or 0.1, 1, 10, and 100 μg/mL of EEEG for 24 h, followed by ox-LDL loading. The result showed (Figures 1B,C) that EEEG significantly reduced macrophage foam cell formation compared with vehicle control. Similarly, BODIPY staining showed that EEEG (1 and 10 μg/mL) significantly inhibited ox-LDL-induced intracellular lipid droplets accumulation in RAW264.7 macrophages (Figures 1D,E). These results suggested that EEEG effectively inhibited minimally modified lipid accumulation and macrophage foam cell formation.

### EEEG treatment reduces macrophage uptake of ox-LDL

**Figure 2:**
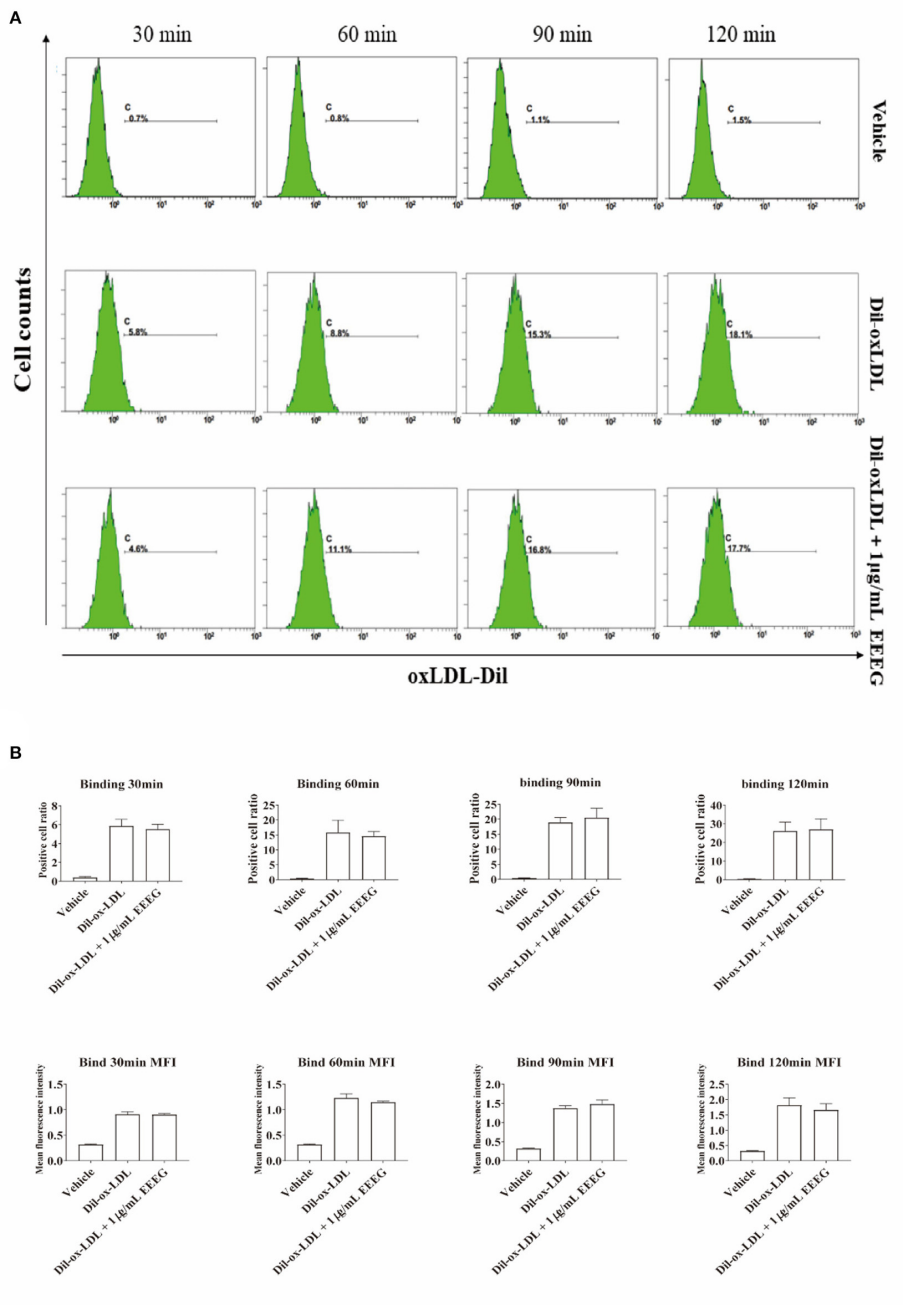
EEEG treatment does not influence the surface-binding of oxLDL to macrophages. **(A)** Representative histogram plots of Dil-ox-LDL binding by macrophages. Cells were incubated for 30, 60, and 90 min with Dil-ox-LDL in the presence or absence of EEEG, followed by flow cytometry. **(B)** Quantification of the percentage of positive cells and mean fluorescence intensity values. DiI-ox-LDL-fluorescence is shown on the Y-axis and macrophages on the X-axis. Data are expressed as mean ± s.e.m., Statistical analysis was based on Graphpad Prism 9.0.2 software and a value of *P* < 0.05 was considered statistically significant. One-way ANOVA with Student Neuman-Keuls *post-hoc* test was performed to compare the data between multiple groups.

**Figure 3:**
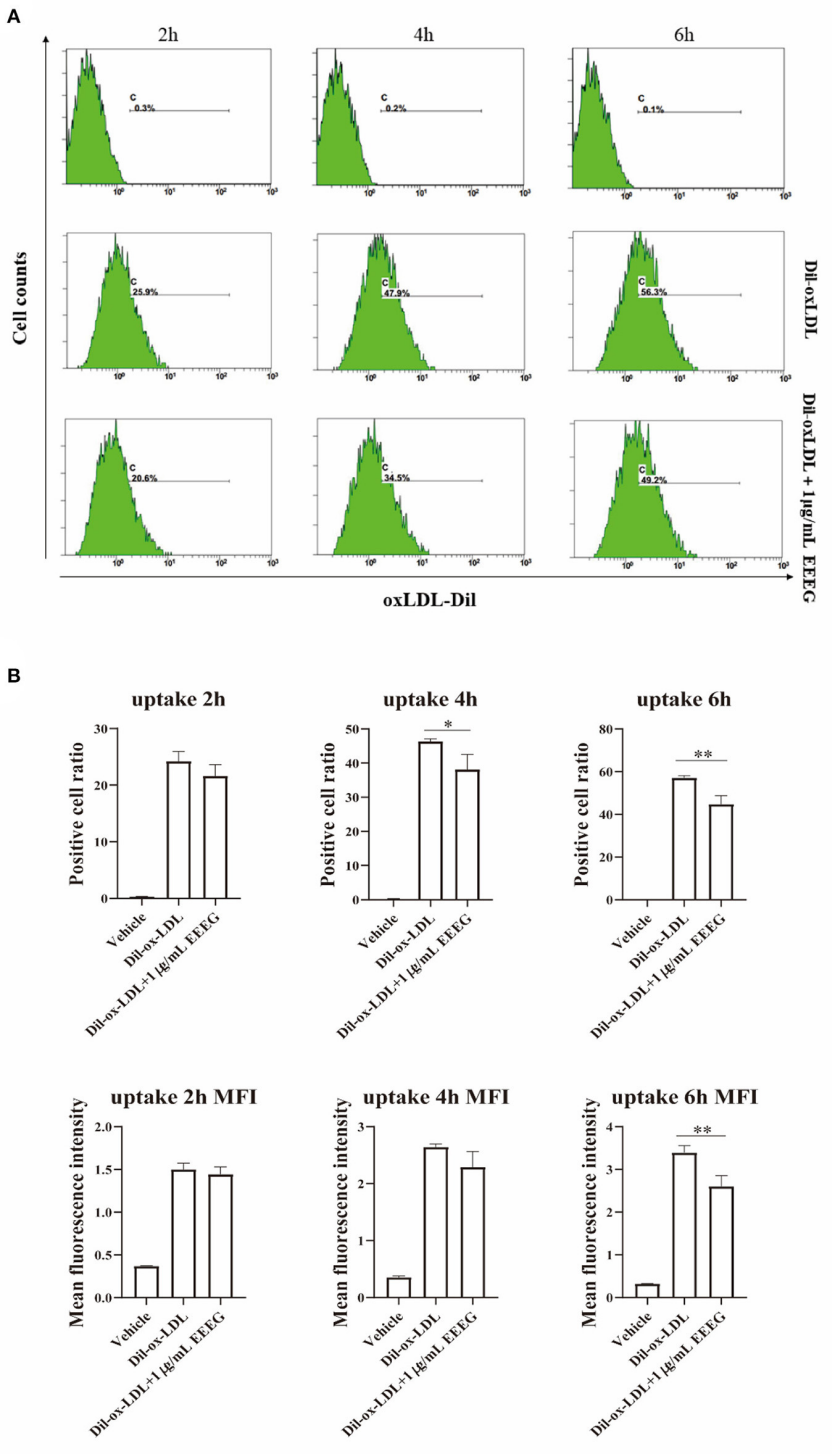
EEEG inhibits the uptake of oxidized low-density lipoprotein by macrophages. **(A)** Representative histogram plots of Dil-ox-LDL binding by macrophages. Cells were incubated for 2, 4, and 6 h with Dil-ox-LDL in the presence or absence of EEEG, followed by flow cytometry. **(B)** Quantification of the percentage of positive cells and mean fluorescence intensity values. DiI-ox-LDL-fluorescence is shown on the Y-axis and macrophage numbers on the X-axis. Data are expressed as mean ± s.e.m., Statistical analysis was based on Graphpad Prism 9.0.2 software and a value of *P* < 0.05 was considered statistically significant. One-way ANOVA with Student Neuman-Keuls *post-hoc* test was performed to compare the data between multiple groups, *p < 0.05, **p < 0.01 vs. ox-LDL.

The intracellular cholesterol transport is essential for maintaining cholesterol homeostasis and depends on cholesterol binding, uptake, and efflux (13). To determine which biological processes are affected by EEEG during the foam cell formation, cholesterol binding and uptake were evaluated by flow cytometry. As shown in Figures 2A,B, compared to vehicle control, EEEG (1 μg/mL) treatment did not change the surface Dil fluorescent intensity after Dil-oxLDL incubation up to 120 min. This result indicated that EEEG did not affect cholesterol binding in macrophages. Next, BMDMs were treated with Dil-oxLDL+EEEG for 2, 4, and 6 h, respectively. The results showed that compared with the Dil-oxLDL group, the number of positive cells in the Dil-oxLDL+EEEG group was significantly reduced at 4 and 6 h. The mean fluorescence intensity of the Dil-oxLDL+EEEG group was also significantly lower than that of the Dil-oxLDL alone group at 6 h (Figures 3A,B), indicating that EEEG reduced macrophage foam cell formation by decreasing uptake of ox-LDL.

### EEEG reduces the expression of genes essential for ox-LDL uptake

**Figure 4:**
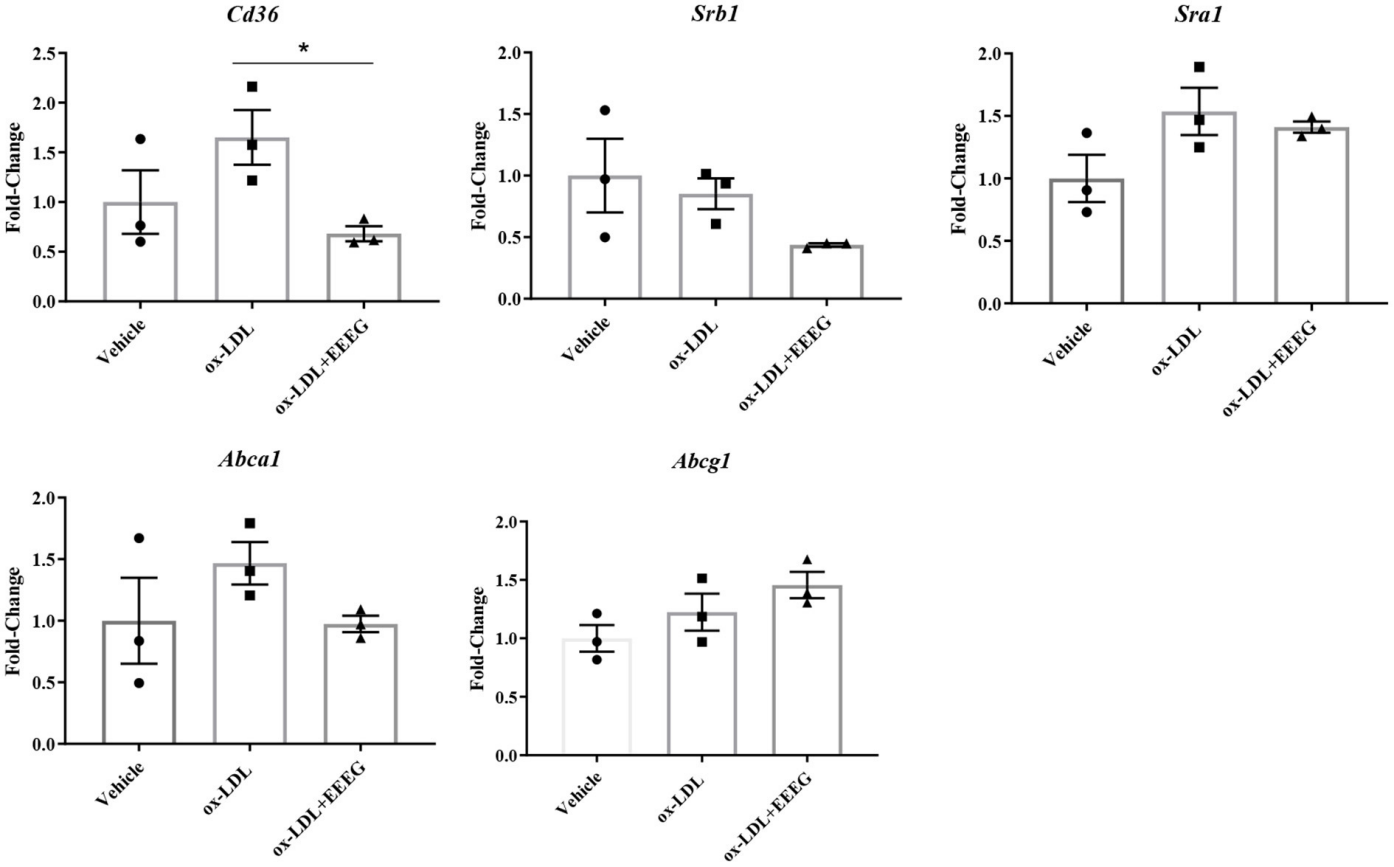
EEEG treatment reduces expression levels of genes involved in ox-LDL uptake. Expression levels of cholesterol homeostasis-related genes involved in uptake and efflux of ox-LDL in BMDM. Data are expressed as mean ± s.e.m., Statistical analysis was based on Graphpad Prism 9.0.2 software and a value of *P* < 0.05 was considered statistically significant. One-way ANOVA with Student Neuman-Keuls *post-hoc* test was performed to compare the data between multiple groups, *p < 0.05.

Scavenger receptors and cholesterol transporters ABCA1 and ABCG1 are essential for cholesterol uptake and efflux (13). To examine the levels of gene expression involved in cholesterol uptake and efflux in BMDM cells before and after EEEG treatment, quantitative RT-PCR was used to detect the mRNA levels of *Cd36, Sra1, Srb1, Abca1*, and *Abcg1*. Consistent with the previous results (Figures 3A,B), the expression levels of *Cd36* was significantly decreased after EEEG treatment (Figure 4). There was no significant difference in the expression of *Sra1, Srb1, Abca1*, and *Abcg1* mRNA expression in the EEEG group compared with those in the vehicle control group. These results strongly suggested that EEEG may reduce the formation of foam cells by inhibiting cholesterol uptake but not cholesterol efflux *via* down-regulating scavenger receptors' expression levels of CD36. To evaluate whether the inhibitory effect of EEEG on foam cell formation was dependent on CD36. THP-1 cells were treated with PPAR-γ agonist, Rosiglitazone (RSG), PPAR-γ is essential for basal expression of CD36 (14), and the result showed a significant increase in lipid droplets in the ox-LDL + RSG group compared with ox-LDL group. After treatment with 1 μg/mL of EEEG, a significant reduction in lipid droplets was observed in the ox-LDL + RSG + EEEG group compared with ox-LDL + RSG group (Supplementary Figure 2), indicating that reduction of uptake of ox-LDL by EEEG may be dependent on CD36.

### EEEG treatment attenuates atherosclerotic plaque formation in HFD-fed ApoE^−/−^ mice

**Figure 5:**
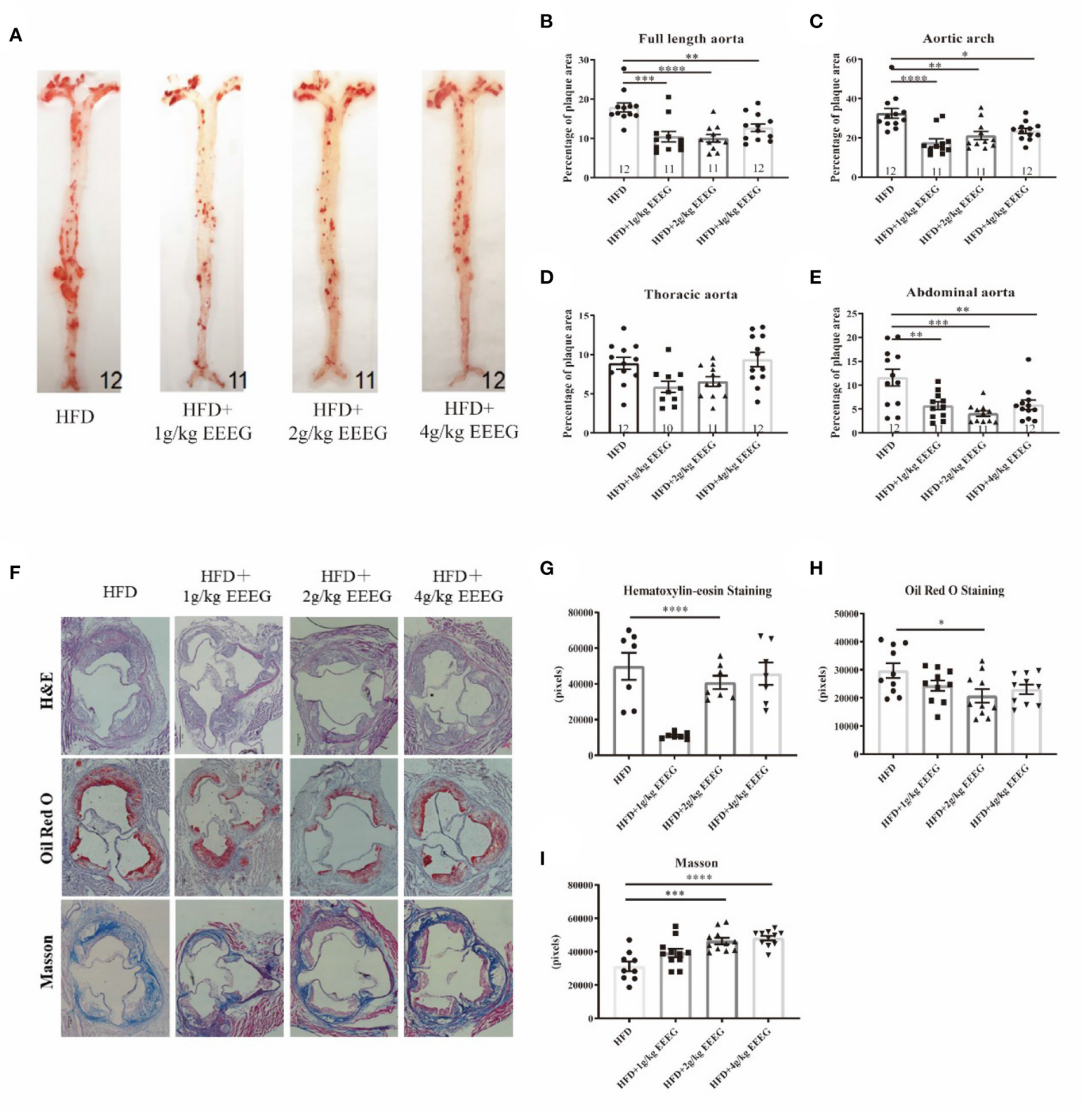
EEEG reduces atherosclerotic plaque areas in HFD-fed ApoE^−/−^ mice. **(A)** Representative images of Oil-Red-O staining of entire aortas including the aortic arch, thoracic, and abdominal regions. **(B–E)** Quantitative analysis of aorta lesion areas stained with Oil-Red-O. **(F)** Representative images of H&E, Oil-Red-O, and Masson staining of aortic root. **(G–I)** Quantitative analysis of aortic root section lesion areas stained with H&E, Oil-Red-O, and Masson. HFD (*n* = 12), HFD+1 g/kg EEEG (*n* = 11), HFD+2 g/kg EEEG (*n* = 11), HFD+4 g/kg EEEG (*n* = 12), Data are expressed as mean ± s.e.m., Statistical analysis was based on Graphpad Prism 9.0.2 software and a value of *P* < 0.05 was considered statistically significant. One-way ANOVA with Student Neuman-Keuls *post-hoc* test was performed to compare the data between multiple groups, *p < 0.05, **p < 0.01, ***p < 0.001, ****p < 0.0001 vs. HFD.

Macrophage foam cell formation is a hallmark of atherosclerosis (15). Since EEEG effectively inhibited lipid uptake and foam cell formation *in vitro*, we thus hypothesized that it might attenuate atherogenesis. To test this hypothesis, ApoE^−/−^ mice were fed with HFD and treated with vehicle, 1, 2, or 4 g/kg of EEEG for 16 weeks. Full-length aorta and aortic roots were collected and stained with Oil red O, hematoxylin-eosin, and Masson's trichrome. As shown in Figure 5, the area of plaques from mice treated with EEEG was significantly lower than in vehicle treated mice. The size of plaques in mice administrated with 1 and 2 g/kg of EEEG was smaller in the aortic arch and abdominal aorta compared with mice fed with the vehicle. Consistently, the plaque area in the aortic roots from mice treated with 1 g/kg of EEEG was also markedly smaller than that in mice fed with vehicle (Figures 5A,B). In addition, collagen fiber contents in the aortic roots of mice treated with EEEG significantly increased as compared to those in mice fed with vehicle (Figures 5C,D). These results indicated that EEEG inhibits atherosclerotic plaque formation and enhances plaque stability in ApoE^−/−^ mice.

### EEEG inhibits macrophage content in atherosclerotic plaque

**Figure 6:**
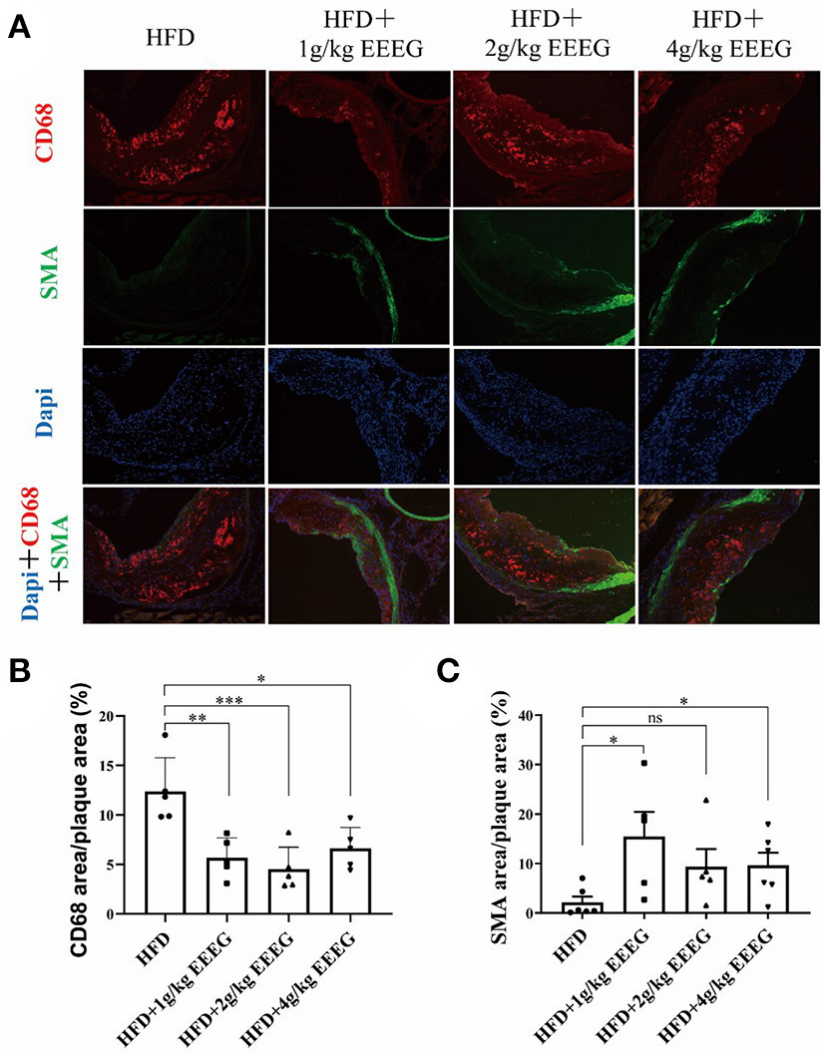
EEEG treatment significantly inhibits macrophage infiltration in the aortic root of mice. **(A)** Representative images of CD68 (macrophages, red) and α-SMA (α-smooth muscle actin; green, smooth muscle cells) immunostaining of aortic root sections. **(B,C)** Quantitative analysis of macrophages and smooth muscle cell content in the aortic root sections. HFD (*n* = 5), HFD+1 g/kg EEEG (*n* = 5), HFD+2 g/kg EEEG (*n* = 5), HFD+4 g/kg EEEG (*n* = 5), Data are expressed as mean±s.e.m., Statistical analysis was based on Graphpad Prism 9.0.2 software and a value of *P* < 0.05 was considered statistically significant. One-way ANOVA with Student Neuman-Keuls *post-hoc* test was performed to compare the data between multiple groups, *p < 0.05, **p < 0.01, ***p < 0.001. ns, no significance.

Next, the cellular composition of the plaques was examined by immunofluorescence staining. As shown in Figure 6, treatment with 1, 2, and 4 g/kg of EEEG significantly attenuated CD68^+^ macrophages in aortic roots by ~95.4, 56.2, and 86.1%, respectively, compared to vehicle-treated mice. Interestingly, α-SMA positive cells increased by 725, 256, and 304% in 1, 2, and 4 g/kg of EEEG-treated mice. Together with the collagen staining, as shown in Figures 5F–I, our results suggested that EEEG treatment promoted a more stable plaque phenotype in atherogenic mice.

### EEEG treatment reduces cholesterol absorption in HFD-fed ApoE^−/−^ mice

**Figure 7:**
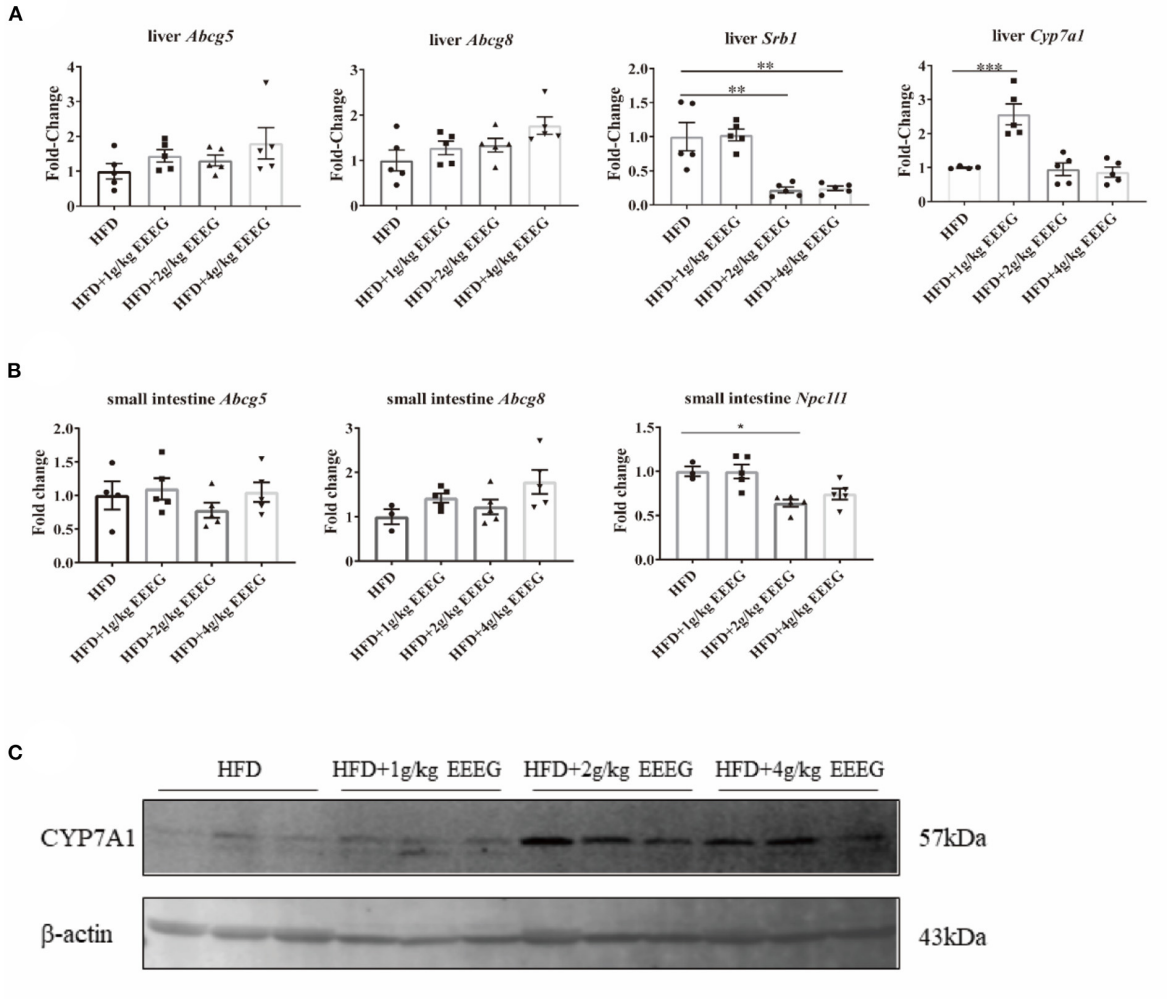
EEEG reduces cholesterol absorption in the liver and small intestine of mice. **(A)** RT-qPCR analysis of *Abcg5, Abcg8, Srb1*, and *Cyp7a1* gene expression in liver. **(B)** RT-qPCR analysis of *Abcg5, Abcg81*, and *Npc1l1* gene expression in the small intestine. **(C)** Expression of CYP7A1 in the liver of each group of mice analyzed by Western blotting. Data are expressed as mean ± s.e.m., Statistical analysis was based on Graphpad Prism 9.0.2 software and a value of *P* < 0.05 was considered statistically significant. One-way ANOVA with Student Neuman-Keuls *post-hoc* test was performed to compare the data between multiple groups, *p < 0.05, **p < 0.01, ***p < 0.001.

As shown in Supplementary Figure 3, EEEG significantly decreased serum levels of LDL-C, but did not change the serum levels of TC, TG, and HDL-C. Reverse cholesterol transport involves removing excess cholesterol from plaque and transporting it to the liver for degradation into bile acids (16). The expression levels of genes essential for cholesterol transport and metabolism in the liver and intestine were detected to examine whether EEEG treatment influenced the cholesterol transport and metabolism in HFD-fed ApoE^−/−^ mice. The results showed that both 2 g/kg and 4 g/kg of EEEG treatment significantly reduced hepatic *Srb1* mRNA expression levels; 1 g/kg of EEEG significantly up-regulated hepatic Cyp7a1 mRNA expression levels compared with the vehicle group. In addition, both 2 g/kg and 4g/kg of EEEG significantly up-regulated hepatic Cyp7a1 protein expression levels compared with the vehicle group (Figures 7A,C). No significant difference in mRNA expression of *Abcg5/8* was observed between the EEEG group and vehicle group (Figure 7A). Furthermore, 2 g/kg of EEEG treatment significantly promoted *Npc1l1* expression in the intestine. EEEG did not change the intestinal *Abcg5/8* mRNA expression levels compared with the vehicle group (Figure 7B). These results showed that EEEG might effectively inhibit cholesterol absorption and promote cholesterol metabolism.

## Discussion

Atherosclerotic cardiovascular disease is the leading cause of death in humans. Statins have significant lipid-lowering effects but are unsuitable for all patients with high cholesterol and can cause serious side effects. The flower of *E. gardneri* (Wall.) Meisn is commonly used in beverages to prevent and treat diabetes and cardiovascular disease in Tibet (9). For the first time, the present study shows that EEEG had an anti-atherosclerotic effect in apoE-deficient mice by restraining macrophage foam cell formation.

Foaming macrophages with subsequent fatty streaks formation contribute to the steady growth of atherosclerotic plaques (4). New drugs that inhibit macrophage-derived foam cell formation have important scientific significance for reducing the morbidity and mortality of the atherosclerotic cardiovascular disease. Various medical plants have been shown to possess anti-atherogenic properties by interfering with foam cell development (17). Some herbal extracts, such as *Allium sativum* or *Ocimum basilicum*, can inhibit foam cell formation in human macrophages by reducing scavenger receptor activity *in vitro* (18, 19). Other extracts, such as *Cassia occidentalis* and *Moringa oleifera*, are tested to hinder the development of foam cells in animals (20, 21). Furthermore, a growing body of evidence has shown that bioactive components of medicinal plants, such as flavonoids, gossypetin, and lycopene, suppress foam cell formation by regulating cholesterol transporter, lectin-like oxidized low-density lipoprotein receptor-1, acyl CoA cholesterol acyltransferase activity, and neutral cholesteryl ester hydrolase activity (22–24). This study demonstrated that the EEEG ameliorated ox-LDL-induced foam cell formation in RAW264.7 cells and bone marrow-derived macrophages. The flower of *E. gardneri* (Wall.) Meisn mainly contains flavonoids, coumarins, phenylpropan, triterpenoids, volatile oils, and other components. Flavonoids are among the key medicinal ingredients found in the flower of *E. gardneri* (Wall.) Meisn (25–27). Therefore, we speculated that flavonoids in the flower of *E. gardneri* (Wall.) Meisn played an important role in inhibiting the formation of foam cells, and further studies are warranted to confirm.

Macrophage cholesterol homeostasis is maintained by balancing the influx and efflux pathways. Cholesterol influx occurs by binding and uptake of neutral and modified lipoproteins mediated by SR-A and CD36. In contrast, cholesterol efflux is regulated by lipid-poor ApoA1 or HDL by ABCA1 and ABCG1, respectively (28). Decreased expression of SR-A and CD36 or increased expression of ABCA1 and ABCG1 block foam cell formation in macrophages (29, 30). Previous studies demonstrate that ox-LDL uptake by macrophages is prevented by herbal extracts, such as *Syzygium cumini* leaf extract, *Rubus coreanus* fruit extract, and medicinal plant decoctions (31–33). Here, we observed the EEEG reduced the uptake of Dil-ox-LDL by macrophages but had no noticeable effect on the binding of Dil-ox-LDL. In accordance with this result, expression of CD36 was markedly decreased, and expression of SR-A tended to be down-regulated by the EEEG. The expression of CD36 is tightly regulated by PPAR-γ in response to the stimuli (34). Therefore, we used the PPAR-γ agonist, Rosiglitazone to evaluate whether the inhibitory effect of EEEG on foam cell formation was dependent on CD36. After treatment with 1 μg/mL of EEEG, a significant reduction in lipid droplets was observed in the ox-LDL + RSG + EEEG group compared with ox-LDL + RSG group, and there was no significant difference between ox-LDL + EEEG group and ox-LDL + RSG + EEEG group (Supplementary Figure 2). The expressions of ABCA1 and ABCG1 were not altered in the macrophages. Collectively, these data implied that the EEEG inhibited macrophage-derived foam cell formation by decreasing uptake of ox-LDL *via* reducing expression of CD36. How EEEG reduces CD36 expression remains to be elucive.

As shown in Figure 5, the EEEG attenuated atherosclerotic plaque size and lipid content (oil-red-O staining) in ApoE^−/−^ mice. EEEG treatment significantly decreased the macrophage-positive area in the aortic sinuses in HFD-fed ApoE^−/−^ mice. Macrophage-derived foam cell formation plays a critical role in the early event of atherogenesis (35). The decreased macrophage-positive area and lipid content suggest EEEG may inhibit inflammation and lipid loading in atherogenesis *in vivo*. Collagen fibers are the main component of atherosclerotic lesions and are used as an index to evaluate plaque stability (36). The EEEG treatment increased the number of vascular smooth muscle cells and the amount of collagen fiber, indicating that the EG may stabilize atherosclerotic plaque. However, its underlying mechanism still needs to be uncovered.

Reverse cholesterol transport is a pathway that transports cholesterol from peripheral tissues to the liver and intestine for excretion (37). *Srb1, Abcg5/8*, and *Cyp7a1* are involved in the uptake of cholesteryl esters, cholesterol excretion, and cholesterol metabolism in the liver, respectively. In contrast, intestinal sterol transporters *Abcg5/8* and *Npc1l1* are involved in the excretion of cholesterol from enterocytes into the lumen and absorption of cholesterol from the lumen into enterocytes, respectively. In this study, the medium and high doses of EEEG decreased the mRNA expression of hepatic *Srb1* and intestinal *Npc1l1*. The low dose of EEEG increased the mRNA expression of hepatic *Cyp7a1*. Together, we inferred from the results that EEEG inhibited the uptake of cholesteryl esters in the liver and intestine and promoted the transformation of cholesterol into bile acid in the liver, which was dependent on the dose of EEEG.

**Figure 8:**
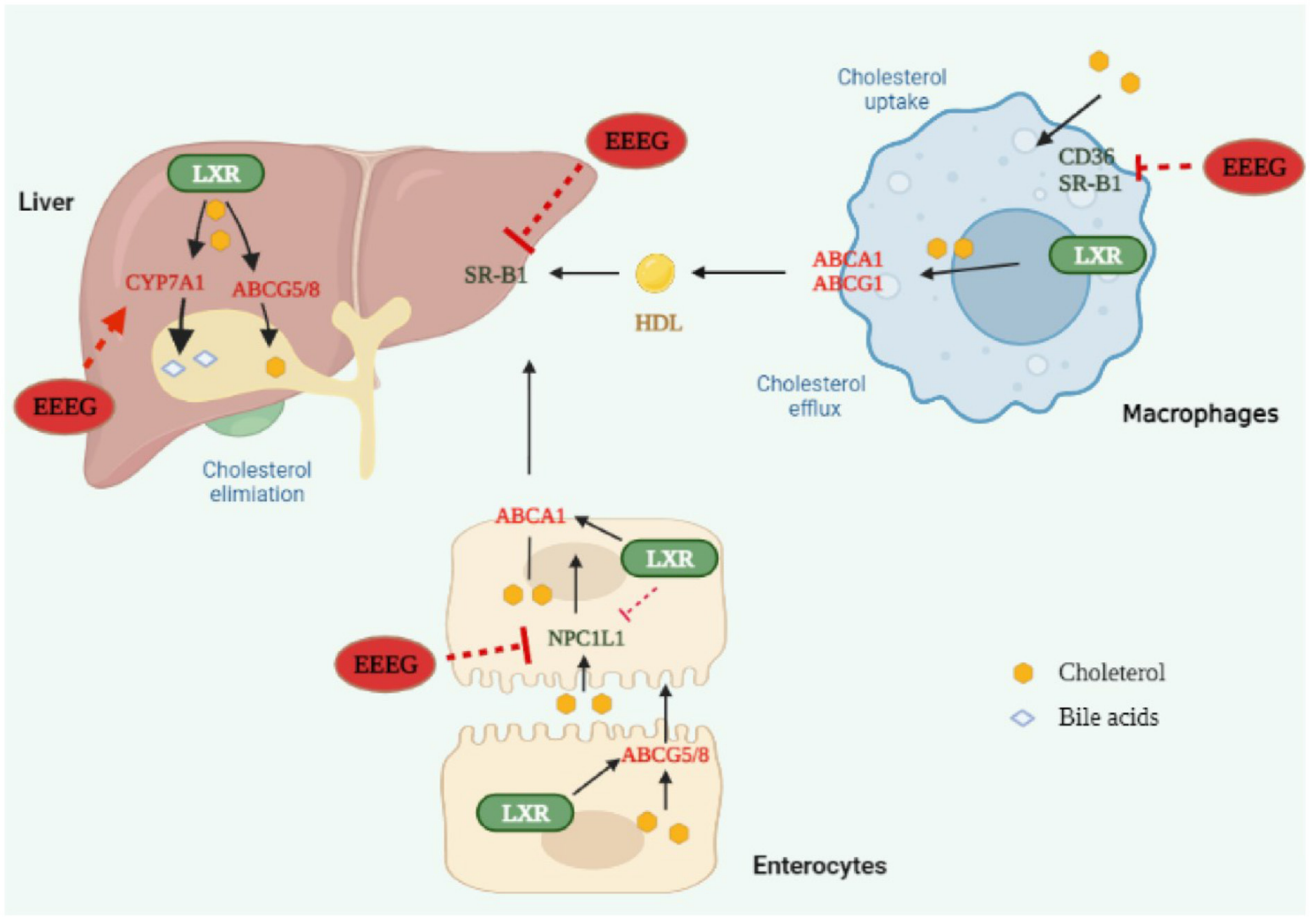
Edgeworthia gardneri (Wall.) Meisn attenuates atherogenesis in mice by inhibiting macrophage foam cell formation via decreasing CD36-mediated ox-LDL uptake. EEEG inhibited macrophage-derived foam cell formation induced by oxidized low-density lipoprotein (ox-LDL) by reducing CD36-mediated lipoprotein uptake. Furthermore, EEEG treatment significantly downregulated mRNA expression of hepatic *Srb1* and intestinal *Npc1l1* and increased expression of hepatic *Cyp7a1*, which may promote the reverse cholesterol transport and decrease the serum levels of cholesterol. In conclusion, EEEG may play a role in attenuating atherosclerotic plaque formation by reducing macrophage foam cell formation.

In conclusion, our study demonstrated that EEEG decreased CD36-mediated ox-LDL uptake and macrophage foam cell formation ultimately inhibited atherosclerosis (Figure 8). This study shed light on understanding the anti-atherosclerotic effect and mechanism of the flower of *E. gardneri* (Wall.) Meisn.

## Data availability statement

The original contributions presented in the study are included in the article/Supplementary material, further inquiries can be directed to the corresponding authors.

## Ethics statement

The animal study was reviewed and approved by Ethics Review Committee of the Jiangxi University of Chinese Medicine.

## Author contributions

LT performed the experiment. CK performed the data analyses and wrote the manuscript. DS, MS, and JL contributed significantly to analysis and manuscript preparation. LQ contributed to the conception of the study. JY helped perform the analysis with constructive discussions. All authors contributed to the article and approved the submitted version.

## Funding

This research was supported by the National Natural Science Foundation of China (82160791 and 81860090), Natural Science Foundation of Jiangxi Province (20202BABL206007), Scientific Research Foundation of the Education Department of Jiangxi Province (GJJ190677), Ph.D. Research Startup Foundation of Jiangxi University of Traditional Chinese Medicine (2019WBZR009), Jiangxi Key Laboratory of Traditional Chinese Medicine for Prevention and Treatment of Vascular Remodeling Related Diseases (20202BCD42014), and Traditional Chinese Medicine Science and Technology Planning Project of Jiangxi Provincial Health and Family Planning Commission (2018B141).

## Conflict of interest

The authors declare that the research was conducted in the absence of any commercial or financial relationships that could be construed as a potential conflict of interest.

## Publisher's note

All claims expressed in this article are solely those of the authors and do not necessarily represent those of their affiliated organizations, or those of the publisher, the editors and the reviewers. Any product that may be evaluated in this article, or claim that may be made by its manufacturer, is not guaranteed or endorsed by the publisher.
