# Supplementary material for: The ethanol extract of Edgeworthia gardneri (Wall.) Meisn attenuates macrophage foam cell formation and atherogenesis in ApoE−/− mice
Source: Front Cardiovasc Med. 2022 Nov 24;9:1023438. doi: 10.3389/fcvm.2022.1023438 (PMC9729707; doi:10.3389/fcvm.2022.1023438)
Supplement: Supplementary file 1 [file Data_Sheet_1.docx]

Supplementary Material

**Supplementary Table 1. Primer sequence**

| Gene | Forward primer (5′-3′) | Reverse primer (5′-3′) |
| --- | --- | --- |
| *Abcg5* | TCTCCGCGTCCAGAACAAC | CATTGAGCATGCCGGTGTAT |
| *Abcg8* | GACAGCTTCACAGCCCACAA | GCCTGAAGATGTCAGAGCG |
| *Npc1l1* | GCTTCTTCCGCAA GATATACACTCCC | GAGGATGCAGCAATAGCCACATAAGAC |
| *Fxr* | TGAGAACCCACAGCATTTCG | GCGTGGTGATGGTTGAATGTC |
| *Srb1* | TAGAGCATATCCCCCAGGTG | GGTACGGGCCAC AAGAAGTA |
| *Cyp7a1* | AGCAACTAAACAACCTGCCAGTACTA | GTCCGGATATTCAAGGATGCA |
| *Lxr* | GCGTCCATTCAGAG CAAGTGT | TCACTCGTGGACATCCCAGAT |
| *β-actin* | GTGGACATCCGCAAAGAC | AAAGGGTGTAACGCAACTA |

# Supplementary Figure 1

Major compounds contained in the 30% EEEG


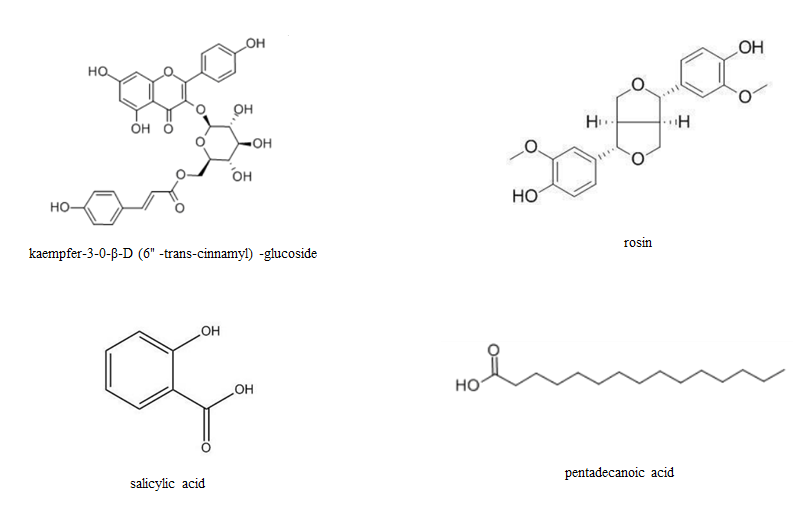


# Supplementary Figure 2


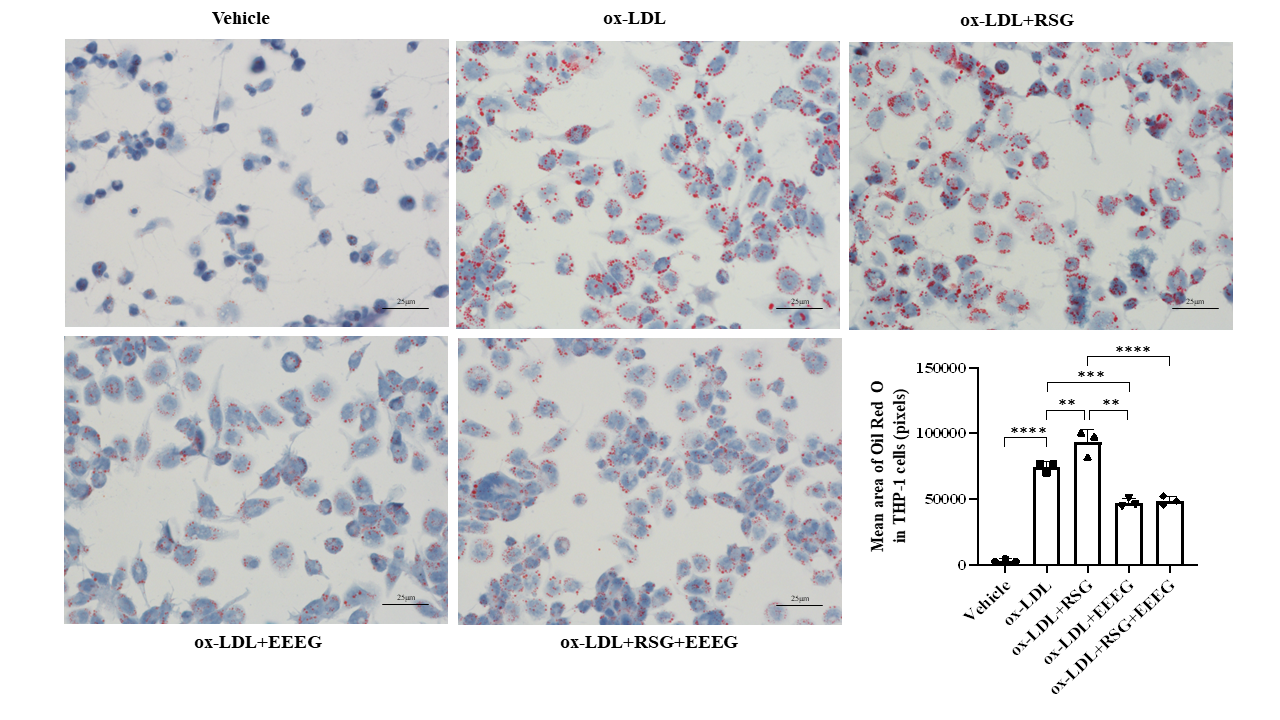


**Supplementary Figure 2. EEEG inhibits macrophage foam cell formation via CD36.** Foam cell formation was determined by Oil-red O staining. Cells were incubated with ox-LDL for 24 h in the presence or absence of 1μM RSG or EEEG, followed by Oil-red O staining. Data are expressed as mean±s.e.m.. Statistical analysis was based on Graphpad Prism 9.0.2 software and a value of *P* < 0.05 was considered statistically significant. One-way ANOVA with Student Neuman-Keuls post hoc test was performed to compare the data between multiple groups, * *p* <0.05, ** *p* < 0.01 *vs* ox-LDL.

# Supplementary Figure 3


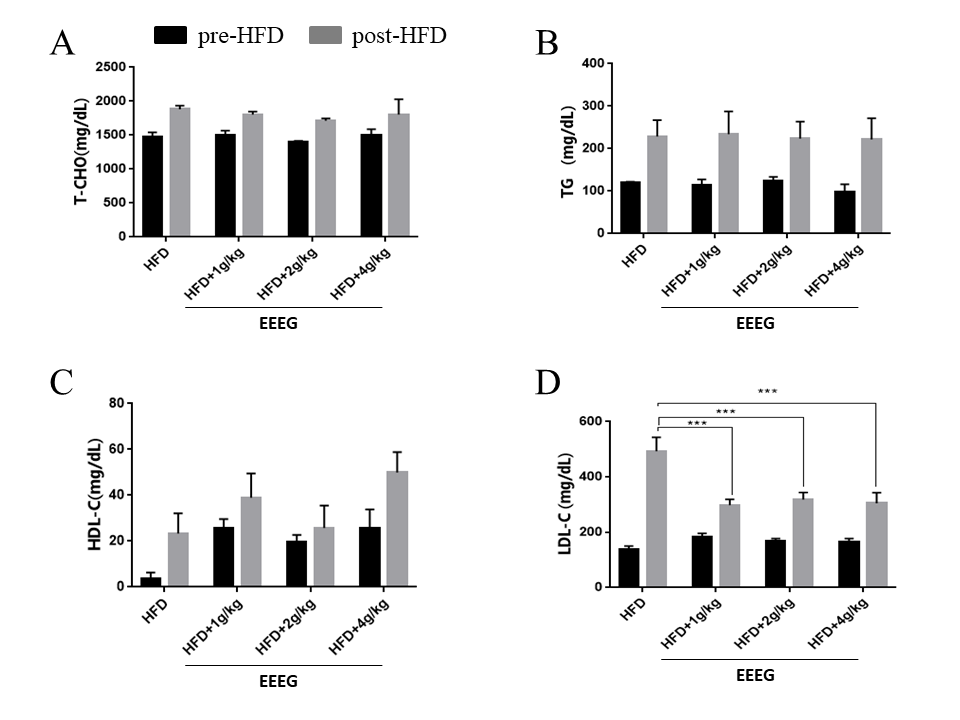


**Supplementary Figure 3. EEEG regulates serum lipid profiles in HFD-induced ApoE^-/-^mice.** A–D Levels of serum lipids (TC, TG, LDL-C, and HDL-C) per group. Data are expressed as mean±s.e.m., Statistical analysis was based on Graphpad Prism 9.0.2 software and a value of *P* < 0.05 was considered statistically significant. One-way ANOVA with Student Neuman-Keuls post hoc test was performed to compare the data between multiple groups. **p* < 0.05, ***p* < 0.01, and ****p* < 0.001 vs. HFD.
